# Optimizing the Design of Oligonucleotides for Homology Directed Gene Targeting

**DOI:** 10.1371/journal.pone.0014795

**Published:** 2011-04-05

**Authors:** Judith Miné-Hattab, Geneviève Fleury, Chantal Prevost, Marie Dutreix, Jean-Louis Viovy

**Affiliations:** 1 Laboratoire Physico-Chimie Curie, UMR CNRS 168, Institut Curie, Paris, France; 2 Laboratoire Recombination, Repair & Cancer, Translational Research Department, Hospital, Institut Curie, Orsay, France; 3 Laboratoire de Biochimie Théorique, CNRS UMR 9080, IBPC, Paris, France; University of Oxford, United Kingdom

## Abstract

**Background:**

Gene targeting depends on the ability of cells to use homologous recombination to integrate exogenous DNA into their own genome. A robust mechanistic model of homologous recombination is necessary to fully exploit gene targeting for therapeutic benefit.

**Methodology/Principal Findings:**

In this work, our recently developed numerical simulation model for homology search is employed to develop rules for the design of oligonucleotides used in gene targeting. A Metropolis Monte-Carlo algorithm is used to predict the pairing dynamics of an oligonucleotide with the target double-stranded DNA. The model calculates the base-alignment between a long, target double-stranded DNA and a probe nucleoprotein filament comprised of homologous recombination proteins (Rad51 or RecA) polymerized on a single strand DNA. In this study, we considered different sizes of oligonucleotides containing 1 or 3 base heterologies with the target; different positions on the probe were tested to investigate the effect of the mismatch position on the pairing dynamics and stability. We show that the optimal design is a compromise between the mean time to reach a perfect alignment between the two molecules and the stability of the complex.

**Conclusion and Significance:**

A single heterology can be placed anywhere without significantly affecting the stability of the triplex. In the case of three consecutive heterologies, our modeling recommends using long oligonucleotides (at least 35 bases) in which the heterologous sequences are positioned at an intermediate position. Oligonucleotides should not contain more than 10% consecutive heterologies to guarantee a stable pairing with the target dsDNA. Theoretical modeling cannot replace experiments, but we believe that our model can considerably accelerate optimization of oligonucleotides for gene therapy by predicting their pairing dynamics with the target dsDNA.

## Introduction

Gene targeting was described in the late 1970s and has since become an important method to achieve stable gene disruption, replacement or modification. This technique is based on the ability of eukaryotic cells to integrate an exogenous DNA molecule into their genome by homologous recombination, HR [1], [2]. It is routinely used to create transgenic mice and has the potential to treat human genetic diseases [3], [4]. This strategy is particularly suitable for curing diseases in which a gene that is necessary for normal cellular homeostasis is inactivated. In this case, correcting the genome of a fraction of the relevant cells is generally sufficient for restoring the metabolic function. Cells have efficient protection strategies to prevent the alteration of their genome by exogenous genetic material, therefore it is important to optimize gene-targeting strategies to overcome this natural defence. The limitations of gene therapy include the internalization of the correcting molecule into cells and into nuclei, the specific recognition of the gene to be repaired, and finally the efficiency of incorporation of the correcting molecule into the host's genome.

One approach used for gene targeting invovles viruses that integrate their genome into the host genome. In these cases the viruses are genetically engineered in order to incorporate the correcting sequence, and to destroy their proliferative ability. This is advantageous because viruses have evolved with their host species, to efficiently solve some of the bottlenecks encountered by gene therapy. However, the viral strategy, while successful in model systems, may lead to potentially deleterious random viral genome integration into the host genome [5]. Virus-based gene therapy also raises the concern that the inactivated virus could recombine with an active one in the host, and create a new infectious virus with unknown and potentially dangerous properties. Consequently, there has been a long-standing interest in developing effective synthetic reagents for gene targeting. In particular, small synthetic oligodeoxynucleotide (ODN) have been used with success by different laboratories [6], [7], [8], [9]. The ability of producing ODNs in large amounts and at low cost is an important advantage of this approach. In addition, it is possible to include specific base modifications in the ODNs in order to prevent their degradation by exonucleases *in vivo*. These modified ODNs are much more stable in the cell than the classical single-stranded DNA molecule [10]. However, experiments involving these new types of vectors suffer from a lack of reproducibility. Delivering these molecules to the cell of interest and incorporating them into the cell and the nucleus is still a challenge, and the overall yield is rather low. Therefore, ODN vectors are used at high doses, raising problems of toxicity and side effects. In addition, even when ODN uptake into the nucleus occurs, the efficiency of correction remains low, due to difficulties in having the correcting sequence finding its homologous target and being substituted into the cellular genome. It is believed that *in vivo*, this process is mainly achieved by homologous recombination through a highly conserved mechanism [1], [2], [11]. Homologous recombination is used by both prokaryotes and eukaryotes to repair DNA damage, to prevent the demise of damaged DNA replication forks, to orchestrate the segregation of homologous chromosomes in meiosis I and in telomere maintenance [12], [13], [14], [15].

The size of the ODN and the position of the heterologous sequences in the ODN affect the efficiency of the correction [16], [17]. However, no consistent rules have been established to optimize ODN design for genetic repair. For any genetic mutation, the number of possible ODNs is large, and optimizing the vector by trial-and-error would be expensive. So far no consistent rules have been established to optimize the design of ODN for genetic repair. It is believed that the homology search, in which the probe “scans” the genome to find its target sequence, is a key step in HR [18]. In a previous article [19], we proposed a Monte-Carlo Metropolis simulation model for the homology search. This model calculates the base-alignment between a probe nucleoprotein filament comprising homologous recombination proteins (Rad51 or RecA) polymerized on a single strand DNA, and a long, target double-stranded DNA. In the present work, we use this algortihm to systematically model the effect of the ODN size and sequence heterology on the efficiency of homology search and strand alignment.

### Description of the strand pairing reaction and principle of the model

Strand pairing is considered to be the rate-limiting step in the process of gene correction by oligonucleotides [7]. Strand exchange is catalyzed by proteins encoded by the *RAD52* epistasis group, which includes the Rad51, Rad52, Rad54, Rad55, Rad57 and RPA proteins involved in HR [20], [12]. Genetic and biochemical studies have shown that RecA and its eukaryotic homolog Rad51 are central enzymes in HR. They polymerize on single-stranded DNA and assemble into helical nucleoprotein filaments [21], [22]. Indeed, it has been shown that over-expression of *RAD51* elevates the frequency of gene targeting [7], [23]. Once homologous sequences are aligned, subsequent strand exchange has been proposed to occur through a concerted rotation of bases within the three-stranded DNA structure [24], [25].

HR proteins were highly conserved in divergent species and the nucleoprotein filaments they constitute on single-stranded DNA exhibit strong similarities. However, the mechanistic details of HR are better established for RecA, the HR protein in *E. coli*. Therefore, we used, when available, molecular constants from this protein in our model. RecA is a 38 kDa protein that polymerizes on single-stranded DNA with a 5′ to 3′ polarity to form helical nucleoprotein filaments with an average DNA spacing of 5.1 A/base. The human homologue, hRad51 forms very similar nucleoprotein filaments with approximately the same average spacing (5.1 A/bp) but it polymerizes on single-stranded DNA with a polarity opposite to that of RecA (3′ to 5′). Both RecA and Rad51 nucleoprotein filaments interact with dsDNA that has a rise of 3.4 A/bp. The difference in base spacing between the ssDNA nucleoprotein filament and the dsDNA is believed to contribute to the homology search in two ways: first, different theoretical studies show that a conformational mismatch between the nucleoprotein filament and its target improves the selectivity of the process [26], [27], [28]. Second, interaction between the nucleoprotein filament and the target leads to a helical structure referred to as synapsis, in which the target is stretched to the same spacing as the nucleoprotein filament. It has been hypothesized that this stretching exposes the target bases promotes base pair exchange [29], [30]. A key question remains: how can sequence information be compared between two molecules in which base pairs are not in register? It has been hypothesized that they can be transiently and locally in register on a few base pairs, thanks to “breathing” of dsDNA by Brownian motion, and to a metastable stretched state of dsDNA with an average spacing close to that of the RecA nucleoprotein filament [31], [32], [33]. This stretched state was clearly identified in single molecule experiments [30], and its structure was studied theoretically [29], [31], [32], [33], [34]. More specifically, simulating a DNA extension of 50% led to a structure strikingly close to that found in the recently solved crystal structure of dsDNA in complex with RecA [29], [32].

This idea of a breathing mechanism of dsDNA is the basis of our model, which is described as follows: the first contact between the nucleoprotein filament and the dsDNA is driven by simple 3-dimensional diffusion and occurs randomly along the duplex (26). The weak non-specific attraction between the nucleoprotein filament and the dsDNA increases the probability of interaction and the trend of the nucleoprotein filaments and dsDNA to align parallel to each other; both increase the efficiency of the homology search [35], [36]. This 3-dimensional Brownian diffusion will not be considered here, since we expect it not to depend on the probe and target sequences and act as a time constant in our results. Our modelling thus starts when the nucleoprotein filament containing the corrector ODN is roughly aligned along the host's chromosome, at a random location. Breathing of the dsDNA creates local zones in which the probe and the target sequences are in register, and sequence comparison is possible. In the presence of homology, a three-stranded structure of lower energy is stabilized, and it can serve as a nucleus to propagate an extended synapsis. If the homology is only fortuitous and local however, the nucleus does not gain energy by extending, and Brownian motion destroys the synapsis. Because of cooperativity, this subtle balance between propagation and detachment of local nuclei is expected to depend strongly on sequence context, and thus to be at the heart of the proofreading properties of HR proteins. We thus investigate in detail how it is affected by local sequence mismatches.

Our model is comprised of different parameters based on experimental information such as the binding energy between the RecA nucleofilament and a targeted dsDNA as well as experimental information on stretched DNA derived from physical studies on single molecule experiments (see method, Supporting Information File S1 and [19] for a more comprehensive description of the model). In Fulconis *et al* [19], we showed that our model accounted for the main features of homologous recombination, such as the detrimental effect of repeated sequences creating a “homology trap”. This effect, investigated both *in vitro* [6] and *in vivo* [37] was well predicted in our model by an important delay in the pairing between two homologous sequences in the presence of repeated sequences [19]. The model also accounted for the presence of a recognition nucleation over a few bases followed by the rapid extension of the synaptic complex, in agreement with previous theoretical studies [26]. Finally, our model predicted that when two or three heterologies are closely clustered, their inhibitory effect on recombination is stronger than when they are far apart, in accordance with *in vitro* experiments [38].

It is important to note that, in this study, we will not directly relate the computed kinetics to a “real” recognition time for two reasons. First, for optimizing ODNs we are mainly interested in a relative comparison between the rates achieved with different ODNs. Second, the model only takes into account the local step of homology i.e the exact alignment of the ODN with target dsDNA: indeed, this step is the only one we expect to depend on the ODN sequence. Several other steps (briefly discussed above) are involved in the complete process, such as the entrance of the ODN into the cell and the nucleus, or its rough diffusion towards the target DNA. Although they should not crucially depend on sequence, these steps involve time constants that typically enter as a multiplicative factor in the final rate.

In this work, we specifically apply the computational model to the search of a target sequence by an ODN containing heterologies. We considered recombination oligonucleotides (ODN) of 25 and 45 bases with mismatches of 1 or 3 bases compared to the dsDNA target. For each size of the ODN and the correcting sequence, 7 different positions of the correcting sequence on the ODN were tested (see method section for the ODN sequences). In each case, we compared the mean time for the ODN to reach a perfect alignment with the target dsDNA, as well as the stability of the complex. Our aim is to understand the effect of the heterology and its flanking sequences on the efficiency of recognition and to provide general rules for optimizing the design of ODNs for gene targeting. We found that inserting a single heterology in an ODN does not affect the stability of the triplex, whereas three consecutive mismatches significantly affect the stability of the triplex. To correct a single heterology, we recommend designing short oligonucleotides (25 bases) in which the mismatch can be placed anywhere in the ODN. In contrast, to correct three consecutive heterologies, we recommend using longer oligonucleotides (at least 35 bases) and inserting the correcting sequence at a central position within the ODN. In general, we recommend using ODN at least **10 times longer** than the size of the correcting sequence; otherwise the presence of “bistable states” in the pairing of the two molecules will be extremely unfavourable to the subsequent steps of recombination.

## Methods

### Fundamental assumptions of the model

**Figure 1:**
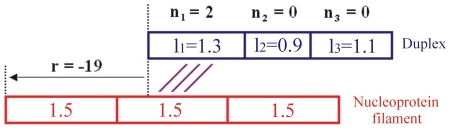
Representation of a dsDNA and its homologous RecA nucleoprotein filament during the simulation. Each rectangle stands for one base (or base pair). All RecA nucleoprotein filament sites always keep the same size, whereas the dsDNA sites' lengths can change (variables *li*). Variable *r* indicates the relative position of the nucleoprotein filament to the DNA. Variable *ni* indicates which DNA site is bound to which RecA nucleoprotein filament site.

A comprehensive description of the model is provided in [19]. Briefly, the fundamental assumptions of the model are the following:

1) The model starts when the nucleoprotein filament containing the corrector ODN is already roughly aligned along the host chromosome at a random position. It does not take into account the “global search” process by 3-dimensional diffusion since we expect it not to depend on the probe and target sequences and act as a time constant in our results. On the contrary, at the local scale, when an ODN and a target chromosome are roughly in contact but not yet in register, homology search is expected to depend significantly on the sequence in particular on the position of the heterology on the ODN. The simulation then calculates the time necessary to have a perfect alignment between the two molecules. To reduce the time of the simulations, we use the same size for the host chromosome and the corrector ODN. Since our model deals with the sequence and length effect during homology search at a local scale, it is relevant to consider only the part of interest of the host chromosome.

2) At the local scale, homology search is considered to occur by a one-dimensional random walk of one molecule relative to the other. It is also considered bidirectional i.e without sliding between the two molecules in a specific direction as confirmed experimentally by Adzuma [39]. Therefore, we shall not take into account the polarity of the single-stranded probe. Moreover, the target dsDNA is considered nucleosomes free and linear at the site where HR occurs. Indeed, even if DNA repair occurs in a chromatin context, nucleosomes remodeling is now recognized as an important regulatory feature by allowing repair factors access to damaged sites.

3) Possible changes of interaction energies associated with ATP hydrolysis are not taken in account. ATP hydrolysis is expected to play a role in the release of the exchanged duplex, but not in the recognition step under scrutiny here [40].

4) The ssDNA inside the nucleoprotein filament is assumed to have a regularly distributed interbase spacing equal to 1.5 times that of canonical B-DNA (Fig.1 and Supporting Information File S1). This regular distribution may appear to contradict the most recent structural information on the stretched structure of RecA bound DNA [41]. Indeed, in this structure, both the single-stranded DNA and the dsDNA resulting from strand exchange present irregular base spacing, with B-DNA-like succession of three base levels followed by a stacking interruption. We have tested in a previous work the hypothesis of irregular base spacing within groups of three successive base levels in our Monte Carlo model [19], [42]. In this experiment, Fulconis *et al* introduced a trinucleotide periodicity using different values of homologous binding energy Ebind to the bases in position 1, 4, 7 (noted 1[3], Ebind = −6.25), 2[3] (Ebind5.5) or 3[3] (Ebind = −4.75). The overall recognition time with respect to the same sequence with Ebind = −5.5 for all bases almost did not change (over 100 simulations, the ratio of recognition times was 1.08). The only difference was found when introducing heterologies at position 3[3] (ratio 0.65).

The dsDNA is subject to local compression or over-extension due to longitudinal thermal fluctuations. The dsDNA is divided into N sites. Each site *i* represents one base pair and is characterized by a variable l*_i_* describing its extension compared to the B form. Another set of variables *ni* describes the binding of the dsDNA to the nucleofilament. If site *i* on the dsDNA is not bound to the nucleofilament, then *ni* = 0. Otherwise, site *i* is bound to a site *j* on the filament (*j* = 1, …, N), and we have *ni* = *j* (Fig.1 and Supporting Information File S1).

5) No hypothesis is made about the detailed molecular mechanism involved in recognition at the single base pair level, either triplex interaction or exchanged Watson-Crick bonding: all the base pairs are considered to be free to interact with the bases inside the nucleoprotein filament at a given time. Also, the helical character of the molecules and the need for a mutual intertwining during strand invasion are not considered. This topological problem, which raises interesting challenges for *in vivo* strand exchange, is not considered here due to the small size of the ODNs.

6) The energy involved in dsDNA RecA-ssDNA bonding depends on several factors, namely, weather the bases are homologous or not; how good the longitudinal alignment between the interacting bases is; and what the local extension state of the dsDNA is. To be more precise, we assume that the duplex has to be locally stretched by thermal fluctuations for the bonding to the nucleoprotein filament to be favourable. The expression of the energy of the system is given in the Supporting Information File S1 and [19].

Note that in the model, we focus on the pairing between a correcting sequence and a target dsDNA and will not consider the depolymerization of HR proteins and the release of the exchanged dsDNA after the formation of an extended synapsis. First, this mechanism is not fully understood, in particular in the case of hRad51, in which it could involve other proteins [43]. Second, it is not expected to be the limiting step in HR, and should also not be sequence dependent.

Note that all molecular parameters used in the model are extracted from experiments on dsDNA and RecA. This is of course questionable for an optimization with a final application in mammals, but unfortunately our present knowledge of similar parameters for hRad51 is too incomplete. It will certainly be useful to repeat similar computations when the parameters for hRad51 are known with the same level of accuracy as for RecA. However, considering the strong structural similarity of RecA and Rad51 nucleoprotein filaments, and the fact that the mechanistic strategy of RecA likely relies on intrinsic DNA dynamics [44], we expect that the qualitative conclusions drawn for RecA regarding local sequences differences will also stand for hRad51.

### Simulations realized

Two kinds of simulations were performed in this study:

- To be able to statistically compare simulated recognition times for various sequences or DNA lengths, we repeated simulations 100 times and computed average values. The process of homology recognition was monitored by recording how many bases are correctly paired as a function of virtual time (number of iterations of the software). We then plotted the mean first time (X-axis) at which a certain number of bases are correctly paired (Y axis). Error bars (computed from the standard deviation) are typically 10 to 15% of the mean value (they are omitted in subsequent figures for reasons of clarity). The simulations end when all bases are correctly paired for the first time. Those simulations will be called “***recognition time simulation***” in the following sections.
- We next investigated the stability of the bonds established by the bases correctly paired to the nucleoprotein filament. To investigate in more detail the mechanisms underlying these statistical results, we performed simulations in which a single realization is followed along the time, without any averaging. To investigate the stability of the complexes once the two molecules are correctly paired, these simulations were performed during 220,000 iterations, a time much longer than the time necessary to achieve full pairing. These simulations are named “**stability simulations**” henceforth. They allow us to evaluate the stability of complexes once full pairing is achieved. An “**instability parameter**” is deduced from these simulations: it is defined as (N-p)/N where p is the average number of bases correctly paired during 120, 000 iterations after the first full recognition occurred, and N is the number of bases in the ODN. Thus, the instability parameter is an extensive parameter equal to 0 when the complex is perfectly stable and 1 when it is instable. The standard deviation of the instability parameter quantifies the amplitude of the fluctuations between the RecA-nucleoprotein filament and target dsDNA after they reach the first correct alignment.
- Finally, we developed a “***graphic interface***” to visualize the model. This interface allows us to visualize the dynamics of the two molecules moving along each other during the homology search. A graphic representation of the simulation is shown in Supporting Information File S1 (Figures S1 and S2), and Supporting Information File S1 (Movies S1 and S2 for a 25 bases ODN and 45 bases ODN respectively). Many aspects of the homology search process remain unclear largely because visualization at the single molecule level is lacking. The development of a graphic interface based on numerical simulation allows us to shed light on certain aspects of the search for homology process, as will be discussed below.

### Sequences used

*In vivo* recombination measurements of phage-plasmid in *E.coli* have revealed that for recombination to be efficient, the length over which homology extends must lie above a minimal value, the MEPS (“minimal efficient processing segment”) [45], [46]. For a completely homologous segment, at least 20 bases are required for significant recombination. Recent results obtained *in vitro* using FRET technique confirmed this minimum size [38]. The CPU time necessary to perform a simulation increases sharply with the ODN size, and with our computation equipment we were practically limited to ODNs of up to 50 bases. Here, we ran simulations using ODN 25 bases and 45 bases long. To maximize biological relevance and facilitate comparison with experiments, we used sequences belonging to the mouse gene of tyrosinase, which provides a convenient *in vivo* model, and was already studied experimentally [6]. The most frequent mutation on this gene associated to the albino phenotype is a G to C transversion at the position 390 (in bold). This mutation, which causes the replacement of the cysteine 85 by a serine can be repaired using an ODN as following:

Albino tyrosinase sequence:

5′CAGGCAACTTCATGGGTTTCAACTGCGGAAACT**C**TAAGTTTGGATTTGGGGGCCCAAATTGTACAGAGA 3′

3′GTCCGTTGAAGTACCCAAAGTTGACGCCTTTGA**G**ATTCAAACCTAAACCCCCGGGTTTAACATGTCTCT 5′

ODN: 3′ 3′TACCCAAAGTTGACGCCTTTGA**C**ATTCAAACCTAAACCCCCGGGT 5′

Corrected sequence:

5′CAGGCAACTTCATGGGTTTCAACTGCGGAAACT**G**TAAGTTTGGATTTGGGGGCCCAAATTGTACAGAGA 3′

3′GTCCGTTGAAGTACCCAAAGTTGACGCCTTTGA**C**ATTCAAACCTAAACCCCCGGGTTTAACATGTCTCT 5′

**Figure 2:**
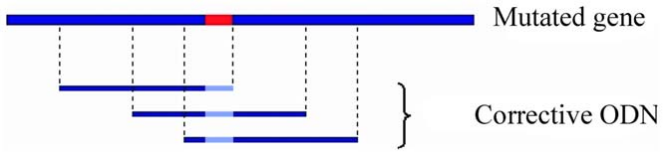
Principle of the simulations. A dsDNA contains a mutation (in red); we simulated the search for homology between a corrective ODN containing the correction (in grey) and the mutant dsDNA.

A corrector ODN with a given size that encompasses the mutation can be constructed at different positions along the gene (Fig. 2). To investigate the effect of this choice on the efficiency of targeting, seven different heterology positions were tested in an ODN of 25 bases: in each case, we analyzed the influence of the heterology position on the recognition time and on the stability of the complex formed. The heterology is underlined in red. The sequences of the seven ODN tested are the following:

*1) ODN25:*

ODN25-1 heterology:

a) First position: **C** ATTC AAAC CTAA ACCC CCGG GTTT

b) Second position: TTGA **C** ATTC AAAC CTAA ACCC CCGG

c) Third position: GCCT TTGA **C** ATTC AAAC CTAA ACCC

d) Fourth position: TGAC GCCT TTGA **C** ATTC AAAC CTAA

e) Fifth position: AAGT TGAC GCCT TTGA C ATTC AAAC

f) Sixth position: CCCA AAGT TGAC GCCT TTGA **C** ATTC

g) Seventh position: AGTA CCCA AAGT TGAC GCCT TTGA **C**

We then tested a heterology covering three consecutive base pairs, in order to get information about the influence of the heterology size. The sequences of the seven ODN tested are the following:

ODN25-3 consecutive heterologies:

a) First position: **CTC** ATTC AAAC CTAA ACCC CCGG GT

b) Second position: CTTT **CTC** ATTC AAAC CTAA ACCC CC

c) Third position: ACGC CTTT **CTC** ATTC AAAC CTAA AC

d) Fourth position: GTTG ACGC CTTT **CTC** ATTC AAAC CT

e) Fifth position: CAAA GTTG ACGC CTTT **CTC** ATTC AA

f) Sixth position: TACC CAAA GTTG ACGC CTTT **CTC** AT

g) Seventh position: AG TACC CAAA GTTG ACGC CTTT **CTC**

*2) ODN45:*

To investigate the time dependence as a function of the ODN length, we also ran the simulations on 45 bases ODN. This size corresponds to the size experimentally tested in [6]. In this case, 5 different heterology positions were tested.

ODN45-1-heterology:

First position:

**C** ATTC AAAC CTAA ACCC CCGG GTTT AACA TGTC TCTT CGCT CAGA

b) Second position:

TGAC GCCT TTGA **C** ATTC AAAC CTAA ACCC CCGG GTTT AACA TGTC

c) Third position:

TA CCCA AAGT TGAC GCCT TTGA **C** ATTC AAAC CTAA ACCC CCGG GT

d) Fourth position:

TCCG TTGA AGTA CCCA AAGT TGAC GCCT TTGA **C** ATTC AAAC CTAA

e) Fifth position:

GACG GTCA CGAG TCCG TTGA AGTA CCCA AAGT TGAC GCCT TTGA **C**

We finally tested a three heterologies mutation on 45 bases ODNs: again, this mutation does not correspond to a disease but gives us interesting information about the influence of the heterology size. The sequences tested for 45 bases ODN are the following:

ODN45-3 consecutive heterologies:

a) First position:

**CTC** ATTC AAAC CTAA ACCC CCGG GTTT AACA TGTC TCTT CGCT CA

b) Second position:

GTTG ACGC CTTT **CTC** ATTC AAAC CTAA ACCC CCGG GTTT AACA TG

c) Third position:

TACC CAAA GTTG ACGC CTTT **CTC** ATTC AAAC CTAA ACCC CCGG GT

d) Fourth position:

AGTC CGTT GAAG TACC CAAA GTTG ACGC CTTT **CTC** ATTC AAAC CT

e) Fifth position:

GA CGGT CACG AGTC CGTT GAAG TACC CAAA GTTG ACGC CTTT **CTC**

## Results and Discussion

### Recognition time

**Figure 3:**
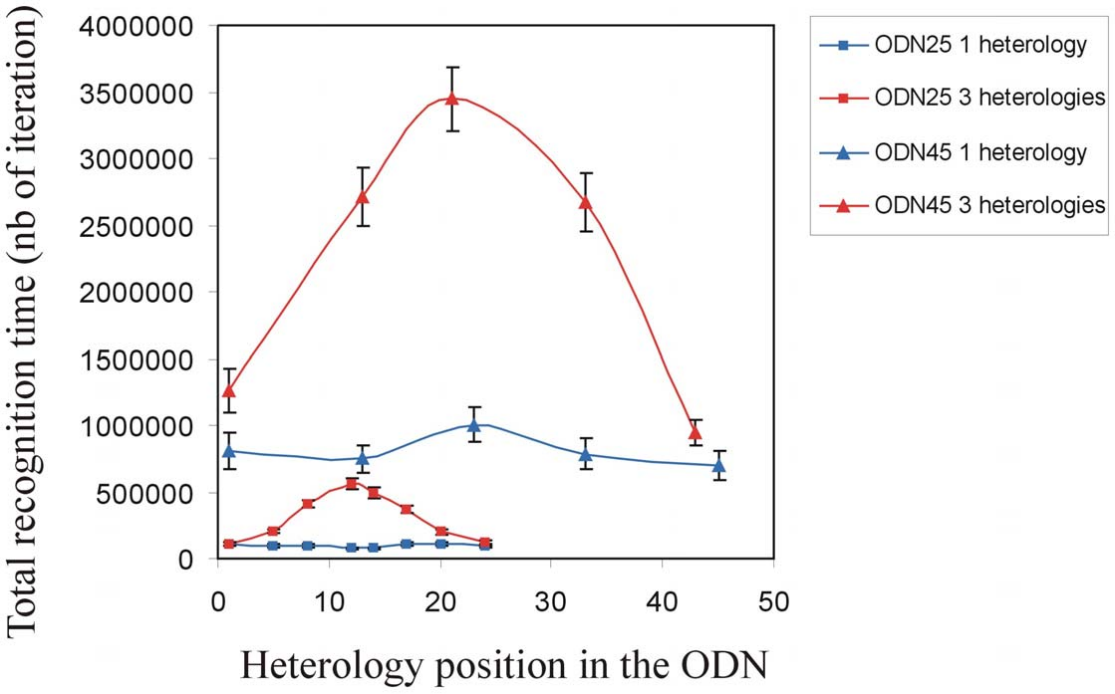
Recognition time as a function of the heterology position. Those graphs were obtained using the “stability simulations” (see the methods section in the text). Blue squares: ODN 25 containing a single heterology; Red squares: ODN 25 containing three consecutive heterologies; Blue triangles: ODN 45 containing a single heterology; Red triangles: ODN 45 containing three consecutive heterologies.

Figure 3 presents the total recognition time for the ODN sequences presented in the method section. For these simulations, we used the “recognition time simulations”, providing an average of the recognition time over 100 simulations. The average recognition time was then plotted as a function of the position of the heterology in the sequence (squares: 25 bases ODN; triangles: 45 bases ODN). The blue curves correspond to the results obtained for the tyrosinase gene mouse mutation containing a single heterology, and the red curves, the results obtained for a mutation involving three consecutive heterologies.

#### Recognition time as a function of the position of the heterology: ODN 25

For an ODN25 containing a single heterology (Fig. 3, blue squared curve), the recognition time does not change as a function of the heterology position. Thus, according to our prediction, the different ODN25 designs have the same efficiency for recognition in terms of timing. In contrast, for ODN25 containing three consecutive heterologies (Fig. 3, red squared curve), the recognition time varies significantly according to the heterology positions, presenting a “bell shape” behaviour. We observed that when three consecutive heterologies are placed at the central part of an ODN25, the predicted recognition time is around 6 times longer than in the case of ODN25 containing a single heterology (Fig. 3, compare the red and blue squared curves). Thus placing three consecutive heterologies in the centre of the ODN is the most unfavourable design in terms of timing of the local homology search.

#### ODN 45

We then performed simulations for 45 bases ODNs containing one or three consecutive heterologies (Fig. 3, triangle curves). First, we observe that the recognition time is much longer for ODN45 than for ODN25 (approximately 7 times longer). Thus, the size of the ODN is a critical parameter to guarantee a fast alignment between the two molecules.

The recognition time varies similarly as a function of the heterology position for ODN25 and ODN45. For both ODN25 and ODN45 containing a single heterology, the recognition time is very similar regardless of the position of the heterology (Fig. 3, blue squares and triangles curves respectively). On the contrary, for ODN25 and ODN45 containing three consecutive heterologies, the recognition time is sensitive to the heterology positions and presents a “bell shape” behaviour (Fig. 3, red square and red triangle curves, respectively).

Those simulations showed that the recognition time is very sensitive to the ODN size. It is around seven times longer for ODN45 than for ODN25. For both ODN sizes containing a single heterology, no particular design is recommended to optimize the recognition time during homology search. Oppositely, for ODN of both sizes containing three consecutive heterologies, central heterologies are the most unfavorable in terms of timing. Thus it is not recommended to place the heterologous zone at the centre to optimize recognition time.

### Stability of the formed complex

So far, we used the “recognition time simulations” allowing us to predict the mean time necessary to obtain the first correct alignment between an ODN and a target dsDNA. However the time necessary for the two DNA molecules to reach a first alignment is not the only relevant parameter for efficient repair. It is also crucial to investigate the stability of the resulting triplex. For that, we performed “stability simulations” using the same sequences (see materials and methods).

#### Stability of perfectly matched complexes

**Figure 4:**
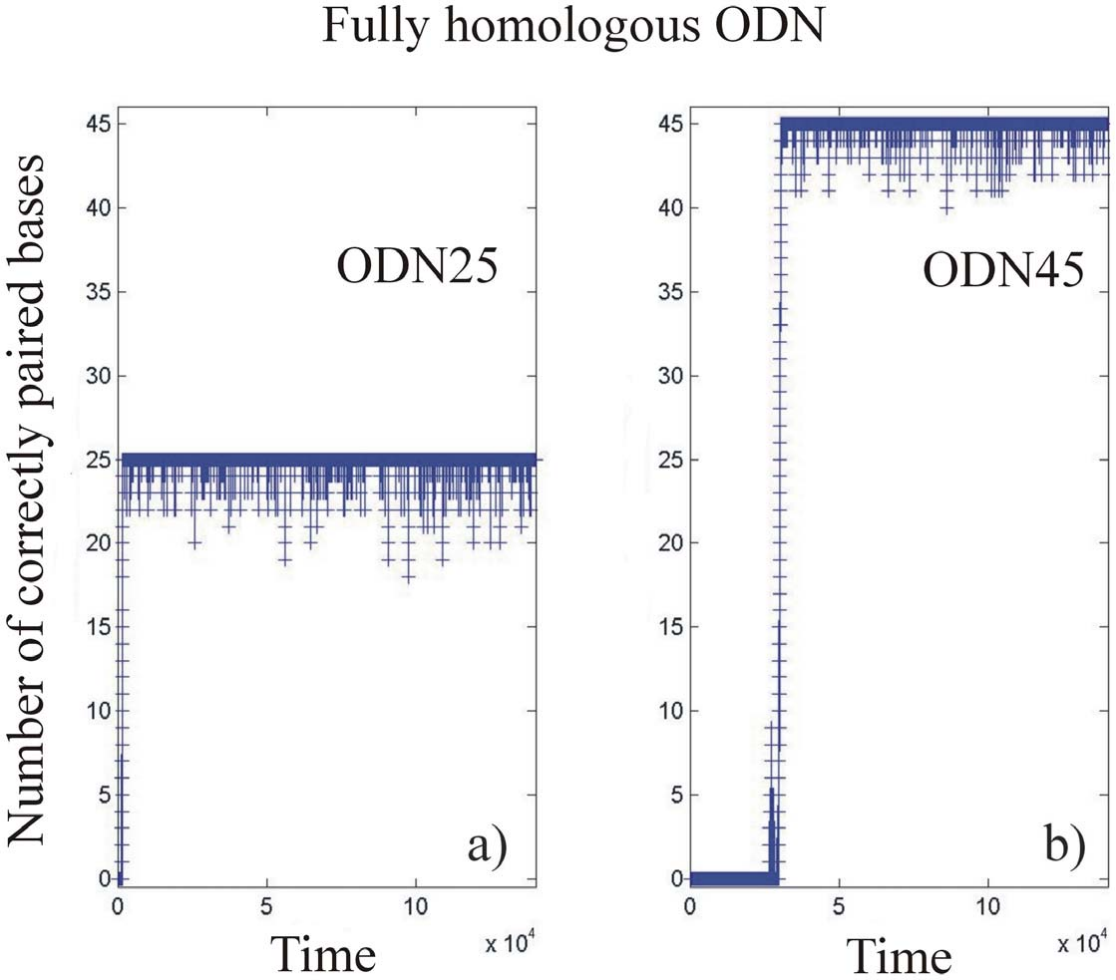
Stability of an ODN containing a complete homologous sequence obtained using the “stability simulations”, i.e that a single realization is followed through the time, without any averaging (see methods in the text). The number of correctly paired bases is plotted as a function of virtual time (number of iterations of the software. a): 25 bases long ODN; b): 45 bases long ODN.

Figure 4a presents the number of correctly paired bases as a function of time for a fully homologous ODN25: those simulations showing the stability between fully homologous DNA are used as a reference. We observe a fast recognition phase; the complex is then globally stable, but continuously undergoes small local fluctuations, the number of correctly paired bases fluctuating between 25 and 19 bases. In this case, the “instability parameter” is quasi null (0.000±0.005) showing that the complex is extremely stable (see Table 1 and materials and methods for a definition of the “instability coefficient”). For fully homologous ODN45, the time necessary for base pairing is significantly increased as shown in Fig. 4b, but the stability of the complex is again extremely high.

**Table 1:** Instability parameter calculated for ODN25 fully homologous and containing one or three heterologies at different positions.

ODN25		
Fully homologous DNA molecules	0.000±0.005	
Heterology position	One heterology	Three heterologies
a)	0.00±0.02	0.09±0.12
b)	0.00±0.02	0.12±0.12
c)	0.000±0.006	0.04±0.12
d)	0.000±0.006	0.04±0.08
e)	0.000±0.006	0.09±0.14
f)	0.00±0.02	0.13±0.15
g)	0.00±0.01	0.13±0.15

The instability parameter is equal to 0 when the complex is totally stable, and 1 when it is totally instable. The standard deviation of the instability parameter quantifies the amplitude of the fluctuations between the RecA-nucleoprotein filament and target dsDNA after the first correct alignment.

**Figure 5:**
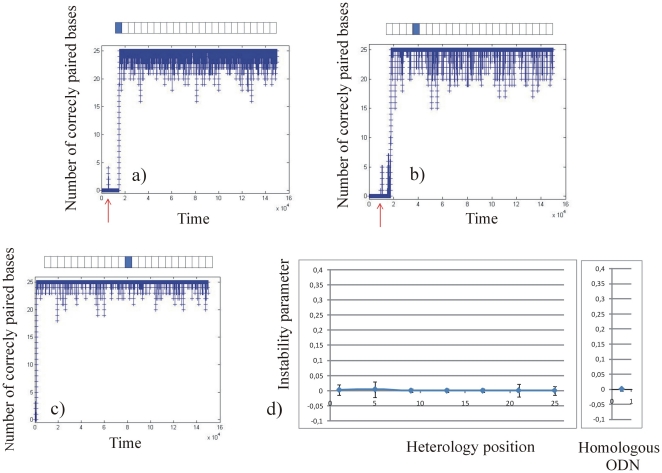
Stability of ODN25 containing a single heterology obtained using the “stability simulations. The number of correctly paired bases is plotted versus the time (number of iterations of the program). Graph 5a: the heterology is placed at the border of the ODN25, Graph 5b: the heterology is placed at an intermediate position. Graph 5c: the heterology is placed at the middle of the ODN. The ODN used for each simulation is represented up to the graph: the heterology is marked in blue. Graph 5d represents the stability coefficient as a function of the heterology position. The stability coefficient of a fully homologous ODN25 is represented on the right as a reference.

**Figure 6:**
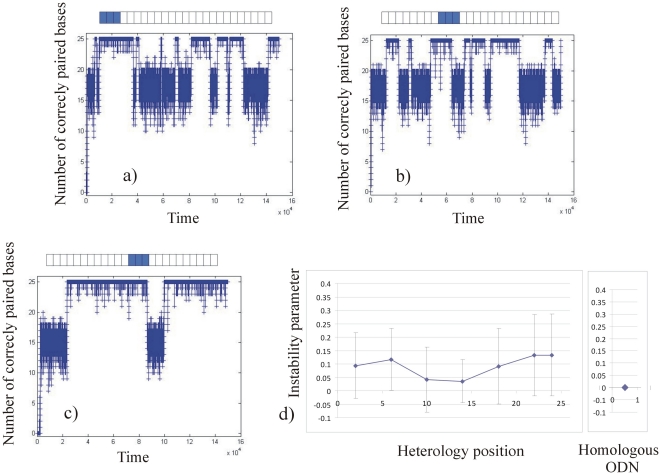
Stability of ODN25 containing three consecutive heterologies obtained using the “stability simulations”. Graph 6a: the heterology is placed at the border of the ODN25, Graph 6b: the heterology is placed at an intermediate position. Graph 6c: the heterology is placed at the middle of the ODN. Graph 6d represents the stability coefficient as a function of the heterologies position. The stability coefficient of a fully homologous ODN25 is represented on the right.

**Figure 7:**
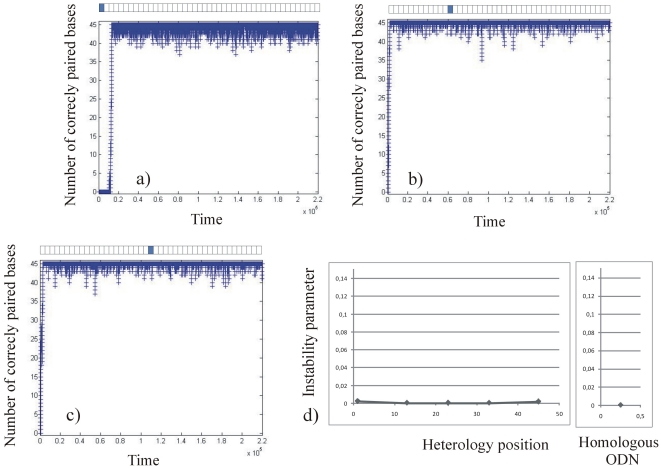
Stability of ODN45 containing a single consecutive heterology. Graph 7a: the heterology is placed at the border of the ODN25, Graph 7b: the heterology is placed at an intermediate position. Graph 7c: the heterology is placed at the middle of the ODN. Graph 7d represents the stability coefficient as a function of the heterologies position. The stability coefficient of a fully homologous ODN45 is represented on the right.

**Figure 8:**
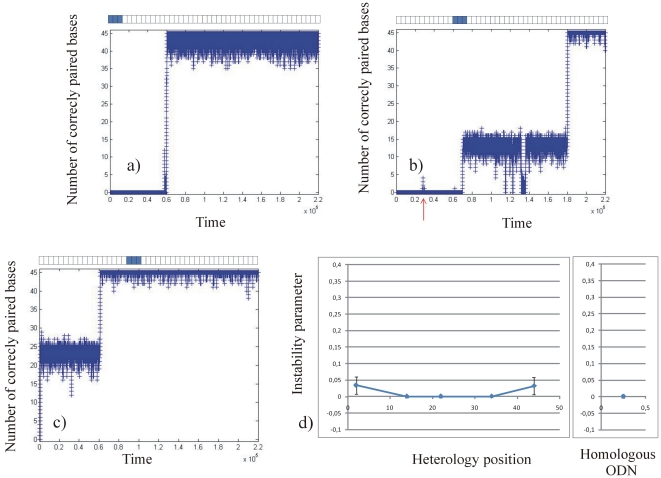
Stability of ODN45 containing three consecutive heterologies. Graph 8a: the heterology is placed at the border of the ODN25, Graph 8b: the heterology is placed at an intermediate position. Graph 8c: the heterology is placed at the middle of the ODN. Graph 8d represents the stability coefficient as a function of the heterologies position. The stability coefficient of a fully homologous ODN45 is represented on the right.

We then ran the “stability simulation” for ODN25 containing a single or three consecutive heterologies (Fig. 5 and 6 respectively), and for ODN45 containing a single or three consecutive heterologies (Fig. 7 and 8 respectively). For the different sequences described in the method section, a graph representing the number of correctly paired bases versus time (number of iterations of the program) was plotted. For clarity, in figures 5, 6, 7 and 8, we present only three typical graphs in which the heterology is placed at one end of the ODN (graphs 5a, 6a, 7a and 8a), at an intermediate position (graphs 5b, 6b, 7b and 8b) and at the middle of the ODN (graphs 5c, 6c, 7c and 8c). Graphs 5d), 6d), 7d) and 8d) display the stability coefficient as a function of the heterology position for all the sequences designed in the method section, as well as the stability coefficient for a perfect homologous ODN as a reference. Note that because we consider that the local search for homology is bidirectional (see methods), the stability coefficient is expected to be symmetrical as a function of the heterology position.

#### Stability of the complex for mismatched ODN25

For ODN25 containing a single heterology, the stability of the complex is very similar to that of a fully homologous ODN25, and the number of correctly paired bases varies from 25 to 15 (see Fig. 5a, 5b and 5c). Figure 5d and Table 1 present the instability parameters of the complex as a function of the heterology position in ODN25. In the different configurations, the instability parameter remains close to 0 showing that the complex formed by the RecA nucleoprotein filament and the dsDNA is very stable whatever the position of the single heterology is. Note that when the heterology is placed at the middle of the ODN25, the instability parameter of the complex is similar to that obtained for fully homologous ODN25 (0.000±0.006, see heterology positions c), d), e) and fully homologous ODN25 in Table 1).

If a single heterology does not significantly affect the stability of the complex, the effect of three consecutive heterologies in an ODN25 is much more important. Figures 6 a), b) and c) display the number of correctly paired bases in ODN25 containing three consecutive heterologies placed respectively at one end, at an intermediate position, and at the middle of an ODN25. In each configuration, the complex formed by the RecA nucleoprotein filament and the dsDNA presents large fluctuations and “bistable states”. Bistable states correspond to a situation in which the two molecules oscillate between a “totally paired state” and a “partially paired state” (Fig. 6a), b) and c)). The frequency of these oscillations is lower when the three heterologies are placed in the middle of the ODN25 (Fig. 6c)) indicating a relatively better stability of the complex in this configuration. Concretely, we observed that in ODN25 with three consecutive heterologies, the RecA nucleoprotein filament and the target dsDNA would never be paired in a stable way. The presence of those bistable states might be extremely unfavourable for the next steps of homologous recombination i.e. the strand exchange.

Figure 6d) and table 1 present the instability parameters as a function of the heterology positions. Figure 6d) presents a clear “inverted bell shape” showing that the stability of the complex is significantly lower when the three heterologies are placed at one of the extremities of an ODN25. Moreover, the standard deviation of the instability parameter is 6 to 20 times higher in the presence of three heterologies than a single heterology (compare left and right columns of Table 1): this means that the fluctuations of the pairing between the RecA nucleoprotein filament and the dsDNA are 6 to 20 times higher in the presence of three heterologies than a single heterology. Overall, the presence of three consecutive heterologies in ODN25 creates bistable states that largely reduce the stability of the complex compared to that observed in the presence of a single heterology.

#### Stability of the complex for mismatched ODN45

Finally, we ran “stability simulations” with 45 bases sequences containing one or three heterologies placed at different positions (Fig. 7 and 8 respectively). Figures 7a), 7b) and 7c) present the number of correctly paired bases when a single heterology is placed at the border, at an intermediate position, and at the middle of an ODN45, respectively. As for ODN25, the pairing between the two molecules is not significantly affected by the presence of the single heterology in the ODN. The instability parameter is very similar for the different configurations to that obtained for a fully homologous ODN45 (see Table 2, left column and Fig. 7d)).

**Table 2:** Instability parameter calculated for ODN45 fully homologous, and containing one or three heterologies at different positions.

ODN45		
Fully homologous DNA molecules	0.000±0.003	
Heterology positions	One heterology	Three heterologies
a)	0.00±0.01	0.03±0.02
b)	0.000±0.004	0.000±0.003
c)	0.000±0.004	0.000±0.003
d)	0.000±0.003	0.000±0.003
e)	0.00±0.01	0.03±0.02

In the presence of three consecutive heterologies in ODN45, the instability parameter varies between 0.03±0.02 in the less stable configuration (heterologies placed at one of the extremities of the ODN45) and 0.000±0.003 in the most stable configuration (heterologies placed in the middle) (see Table 2, right column). Thus, the presence of three consecutive heterologies does not dramatically affect the stability of ODN45 contrary to ODN25. Interestingly, we observe different kinetics of pairing according to the position of the heterologous zone (Fig. 8a, 8b and 8c). When the heterologies are placed at one end of the ODN45, we observe the usual kinetics with a lag time followed by a fast pairing phase (Fig. 8a). When the heterologies are placed at the third or at the middle of the ODN45, we observe “metastable states”: those “metastable states” are characterized by a long and quasi irreversible plateau showing that during a certain lag time, only a part of the two DNA molecules is correctly paired (see Fig. 8b and 8c). In contrast with the “bistable states” observed previously with ODN25 containing three consecutive heterologies, the “metastable states” are long and very rarely reversible. Moreover, once the heterologous zone is crossed, the stability of the complex is similar to that of fully homologous ODN45 (with an instability parameter of 0.000±0.003, see Fig. 8d and Table 2). This confirms the idea that, when three consecutive heterologies are placed near the centre of an ODN45, they can be crossed and lead to a stable triplex, whereas ODN25 containing three heterologies form an instable triplex with “bistable states” extremely unfavourable to the following steps of homologous recombination. In other words, the presence of three heterologies in ODN25 dramatically affects the stability of the RecA nucleofilament and the target dsDNA by creating bistable states, whereas it does not affect the stability of ODN45.

These results suggest that the ratio between the ODN size and the heterology size is a crucial parameter to ensure the stability of the complex once the two molecules are correctly aligned. To investigate further this effect, we tested the stability of different sizes of ODNs, all containing three consecutive heterologies placed at the centre. The sequences used are the following (the heterologous zone is underlined in red):
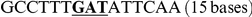


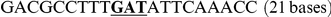


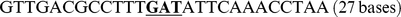


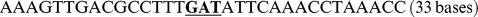


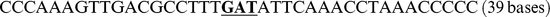


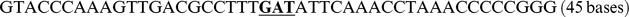



For ODN smaller than 33 bases, we observed “bistable states” similar to those presented in Fig. 6, whereas we observed “metastable states” for ODN longer than 33 bases (data not shown). The presence of “bistable states” is extremely unfavourable for strand exchange; we thus recommend choosing ODN that presents “metastable state” during the pairing even if the mean pairing time is longer in this case. Similar simulations were performed with ODN containing 2 consecutive heterologies: in this case, we observed “bistable states” for ODN smaller than 23 bases and “metastable sates” for ODN longer than 23 bases. Finally, we tested ODN containing 4 consecutive heterologies: ODN smaller than 43 bases presented bistable states whereas longer ODN presented “metastable sates”. From simulations on ODNs containing 2, 3 or 4 consecutive heterologies, we conclude that the ratio between the ODN size and the number of consecutive heterologies should be 10 at least to guarantee a stable binding of the two molecules. In other words, an ODN containing more than 10% consecutive heterologies will never form a stable complex with the target dsDNA.

### Qualitative comparison of our theoretical predictions with *in vitro* and *in vivo* experiments

We compared our theoretical predictions with the existing experiments of gene targeting using ODN in the literature. It is important to point out that comparison between our simulations and *in vivo* experiments is qualitative. Indeed, gene targeting efficiency measured *in vivo* studies allow measuring the final efficiency of the recombination without distinguishing the different steps of gene targeting such as the entrance of the ODN into the cell and the nucleus, the efficiency of homology search and strand exchange. On the contrary, our model focuses on a local step of homology search once the ODN already reached the nucleus and is roughly aligned with the target dsDNA by diffusion. Nevertheless, it is interesting to compare general behaviours of ODN observed *in vivo* with our simulations. One important observation of *in vivo* gene targeting studies using ODN is that large ODNs tend to increase exchange efficiency, ODNs smaller than typically 20 bases being completely inefficient [45], [46]. At first sight, our results on the speed of homology search might seem to be at odds with these results, since 25 base ODNs find their target faster than 45 bases ODNs, whether fully homologous or not. However, we also observed that, in the presence of heterologies, the synapsis formed is unstable, and these instabilities are much more severe for smaller ODNs. Thus, smaller ODNs containing a heterologous zone are faster to find the proper alignment with their target, but weak stability with the target dsDNA prevent them from performing efficient strand exchange. A minimal lifetime is certainly necessary for initiating strand exchange, justifying that the rate of first alignment is not the only relevant parameter for gene correction efficiency. Thus, our results are consistent with *in vivo* experiments. Unfortunately it was not possible, with currently available computing power to probe ODNs larger than 55 bases, and thus to identify more quantitatively the optimal size in this respect. Moreover, it is possible that the higher efficiency of long ODN to perform gene targeting is also due to their much higher probability to reach the nucleus intact than a small ODN.

Numerous *in vitro* studies demonstrated that in ssDNA-dsDNA strand exchange reaction, which normally initiates the recombination process, RecA is able to exchange DNA strands through large heterologies. However, in such cases the efficiency of pairing and strand exchange is significantly reduced [47], [48], [49], [50]. Moreover, it has been shown that RecA protein does not significantly discriminate between perfect and imperfect matches of sequence until the fraction of mismatches approaches 10% [51]. In that *in vitro* study, Bazemore *et al* analyzed the stability of the complex and the rate of strand exchange as a function of the number of heterologies when they are placed at the centre of an ODN. Our simulations are in good agreement with this observation since we predict that an ODN can contain a maximum of 10% consecutive heterologies to guarantee a stable pairing with the target dsDNA. In Jwang *et al* [48], a large heterology placed in the middle or near the middle of a linear duplex yielded the expected product whereas much less of the heteroduplex is seen when the heterlogies are located at either end of the duplex. This is in accordance with our prediction that three heterologies should be placed in the middle or at an intermediate position and not at the edge of an ODN.

### Conclusions

These simulations provide new information on the effects of the position of heterologies in gene targeting: this effect is particularly important for multibase heterologies. The qualitative conclusions of this study are summarized in table 3.

**Table 3:** Summarize of the qualitative conclusions of this study.

	Recognition Time	Stability
**ODN 25 with a one base heterology**	Recognition time not dependent on heterology position.	Good stability dependent on heterology position.
**ODN 25 with a 3 bases heterology**	Recognition time is longer when the heterology is placed at the center of the ODN.	The triplex is never stable whatever the position of the heterologies is (presence of bistable states).
**ODN 45 with a one base heterology**	The recognition time is the same whatever the position of the heterology is.	Good stability whatever the position of the heterology is.
**ODN 45 with a 3 bases heterology**	Recognition time longer when the heterologies are placed at the center of the ODN.	Presence of metastable states: they can be crossed as long as the 3 heterologies are not placed at the edges of the ODN.

**In conclusion, for ODNs containing a single heterology, one should use**

- A short ODN because recognition time will be shorter. As there is only one heterology, there is no risk of observing “bistable states” or “metastable states”, as observed with three consecutive heterologies.
- The heterology can be placed anywhere in the ODN.

**For ODN containing three consecutive heterologies, one should use**

- A long ODN, at least **10 times longer** than the number of heterologies, otherwise, the presence of “bistable states” in the pairing of the two molecules will be extremely unfavourable to the subsequent steps of recombination.
- The heterology should be placed at an intermediate position between the end and the middle of the ODN. In this case, we will observe “metastable states” during the pairing, corresponding to the crossing of the heterologous zone, but, due to the size of the ODN, once this energical barrier is crossed, the pairing will be stable. Moreover, 45 bases ODN with heterologies placed at an intermediate position is the best compromise between a fast recognition time and good stability.
- Consistant with *in vitro* experiments [50], our simulations predict that ODN are stable if they contain less than 10% consecutive heterologies.

Let us end with two final remarks: first, our present conclusions could be refined and extended towards larger ODN sizes, by a mere increase in computing power. Thanks to the fast progress of computer technology, this will be easily achieved in the near future. Second, it will certainly be useful to compare these predictions, which remain qualitative, with *in vivo* and *in vitro* experiments involving ODNs designed for this specific purpose. In return, these experiments will allow us to improve the molecular parameters used as inputs in our modelling, and thus to progress towards a more quantitative model. Among these quantitative pieces of information, several parameters in our model (like the energy related to the nucleoprotein filament binding and the cooperativity cost for the binding) should depend at least to some extent on the nature of the bases involved, but due to lack of base-specific information, so far we could only affect them a “binary” energetic scheme (matched or unmatched). Thus, although the conclusions proposed here are still qualitative, and leave experimentalists with still an important optimization work regarding specific sequence choices, we believe that progress towards more predictive simulations will be possible thanks to interplay between modelling and experiments.

## Supporting Information

File S1 Supplementary Data and Supplementary Material and Methods.
(0.04 MB DOC)

Figure S1 Excerpts of movie 1 showing the local search for homology between a dsDNA and a nucleoprotein filament. The molecules are 45 bases long. Each base of the molecules is represented by a rectangle, as in Fig. 1. The bases are represented in white when they are not in register, in red when they are in register with a non homologous base and in blue when they are in register with a homologous base.
(0.13 MB PNG)

Figure S2 Extracts of movie 2 showing the local search for homology between a dsDNA and a nucleoprotein filament. The molecules are 25 bases long. We used the same colour codes as Fig S1.
(0.24 MB PNG)

Movie S1 Visualization of the search for homology between two complete homologous 25 bases ODN. The recognition occurs preferentially from one of the molecule's extremities. We used the same colour codes as Fig S1.
(0.48 MB MOV)

Movie S2 Visualization of the search for homology between two complete homologous 45 bases. The recognition occurs from a random position of the molecule in 20% of the cases analyzed. We used the same colour codes as Fig S1.
(0.89 MB MOV)
