# Supplementary material for: Optimizing the Design of Oligonucleotides for Homology Directed Gene Targeting
Source: PLoS One. 2011 Apr 5;6(4):e14795. doi: 10.1371/journal.pone.0014795 (PMC3071677; doi:10.1371/journal.pone.0014795)
Supplement: File S1 — Supplementary Data and Supplementary Material and Methods. (0.04 MB DOC) [file pone.0014795.s001.doc]

**SUPPLEMENTAY**

**Supplementary data:**

**Visualization of the search for homology:**

To complete our understanding of local homology search, we realized a “graphic interface” to visualize the motion of the RecA nucleofilament and the target dsDNA predicted by the simulations. From the observation of 15 different movies, we extracted the following general behaviour:

- The search for homology can be described as a two steps process including a nucleation and growth phase (Supplementary Movie 1 and 2). The nucleation phase corresponds to the test for homology and the formation of a transient synapsis. This phase is reversible, with fast binding and unbinding events, as can be clearly observed for instance in Figures 5a), b), and 8b) (see red arrows) and Figure 9. Once several bases are correctly paired, the synapsis is stabilized: the next binding events then progress extremely rapidly in a growth phase (see Movie 1 for ODN25 and Movie 2 for ODN45). From the simulations, we evaluated the size of the synapsis during homology testing prior to extension to 3 ± 1 bases. This corresponds exactly to the value deduced from analytical modelling, 3 ± 1 (25).
- If the ODN contains one or several heterologies, we observe that the nucleus is always formed from a homologous zone; the heterologous zone is then crossed during the growth phase.

- The formation of an extended synapsis always occurs from a single nucleus in our ODNs. Among 15 movies analysed, we never observed homologous recognition from two different points of the molecules at the same time. Indeed, when a “false homology” is found, unbinding occurs before the formation of a second binding event elsewhere because the lifetime and the size of the synapsis are small enough to prevent multi-binding events. In other words, when a “true homology” is found, the growth is fast enough to occur before the formation of a second link elsewhere on the molecules. The fact that the nucleation phase is much slower than the growth phase prevents the formation of discontinuous synapses. It is important to keep in mind that these observations are true within the context of our model and for small ODN of 25 and 45 bases long. Of course, the situation may be different for molecules several tens of thousands bases long.
- For fragments smaller than 30 bases, nucleation almost always occurs at one of the molecule’s extremities (see Movie 1). For fragments longer than 30 bases, the nucleation starts in 20% of the cases from a random point along the molecule (see Movie 2).

Our model involves two cooperative effects: one for DNA stretching (E_coopext_), the other for binding to the nucleoprotein filament (E_coopbind_). Therefore, creating one frontier between a paired segment and an unpaired one is very unfavourable. This is why: i), the progression of correct basepairing is almost always observed to happen on adjacent sites, and not in at random discontinuous sites; and ii), nucleation of the recognition almost always happens at one end of the molecule (which implies creating one frontier only) rather that anywhere else (which implies creating two frontiers). However, this effect would be less pronounced if we dealt with longer molecules, as we observed with 45 bases long ODN.

**Supplementary Material and Method:**

The state of the system is essentially described by extension variables and binding variables. At every time-step in the Monte Carlo procedure, we update one variable: one of the first set of variables at odd dates and one of the second set at even dates.

*Computing the energy:*

The dsDNA is divided into N sites. Each site *i* represent one basepair and is characterized by a variable l*_i_* describing its extension compared to the B form (Fig. 1). Our model is semi-continuous insofar as, for computational efficiency reasons, *l_i_* belongs to the finite set of values {0.7, 0.8, 0.9 …. 1.8, 1.9}. The value *l_i_* = 1 corresponds to the canonical extension *a* = 0.34 nm, whereas *l_i_* = 1.5 corresponds to the base spacing in the nucleoprotein filament. The range of possible extension states, 0.7 · *a* – 1.9 · *a,* is in accordance with the probability distribution for local stretching (31). Another set of variables *n_i_* (*i*=1,…., N) describes the binding to the nucleoprotein filament: if site *i* on the dsDNA is not bound to the nucleoprotein filament, then *n_i_* = 0, otherwise, site *i* of the dsDNA is bound to a site *j* on the nucleoprotein filament, and we have *n_i =_ j* (Fig. 1). The first step in updating the binding variables is to choose a site *i* at random. If *n_i_* ≠ 0, it becomes *n_i_* = 0 (breaking a bond). If *n_i_* = 0, a short algorithm tells us which site *j* of the nucleoprotein filament is just opposite site *i* of the duplex and we impose *n_i_* = *j* (formation of a bond). If there is no nucleoprotein filament site opposite site *i* of the duplex, we keep *n_i_* = 0. Variable *r* indicates the relative position of the nucleoprotein filament to the DNA.

- At time-step t, the system is in a state 1, characterized by variables l_1_,…,L_N_, n_1_, ….,n_N_, r and the energy E_1_.
- At time-step t *+* 1*,* a single variable update is made. This yields a new state 2 with energy E_2_.
- E_1_ and E_2_ are compared. If E_2_ < E_1_, the system goes to state 2 at time t + 1. Otherwise, the probability of transition to state 2 is $e^{\frac{(E_{1}-E_{2})}{k_{B}T}}$, and the probability of remaining in state 1 is the complement to that of the transition probability (k_B_T being the thermal energy). The dependence of the energy on all variables is given by the equation 1 (Fulconis *et al.*, 2005):

$$\begin{matrix} E(l_{1}....l_{N},n_{1}....n_{N})=\underbrace{\sum_{i=1}^{N} E_{ext}(l_{i}}_{1})+\underbrace{\sum_{i=1}^{N-1} E_{coopext}(l_{i},l_{i+1})}_{2} \\ +\underbrace{\sum_{i=1}^{N} (1-\delta(n_{i},0))\times\left(E_{rep}+\alpha(l_{i})(E_{bind}(i,n_{i})-E_{rep})\left(\left(\frac{\sum_{j=1}^{i-1} l_{j}+\frac{1}{2}l_{i}-r-1.5n_{i}+\frac{1.5}{2}}{0.7}\right)^{2}-1\right)\right)}_{3} \\ +\underbrace{\sum_{i=1}^{N-1} E_{coopbind}\delta(n_{i}\times n_{i+1},0)}_{4} \end{matrix}$$

where δ is the Kronecker notation. The meaning of each term is briefly explained below:

1. Energy associated with the extension of the dsDNA. The reference is E_ext_(1)=0, corresponding to the canonical form of the dsDNA. Using the value derived by Cizeau and Viovy, E_ext_(1.7) = 3.75 k_B_T, corresponding to the metastable stretched S-state (30, 32, 39).
2. Energy cost related to the cooperative nature of DNA extension.
3. Energy related to the nucleoprotein filament binding. The central component is E_bind_ (*i, n_i_*), which will take up the value E_hom_ if site *i* on the dsDNA is homologous to site n_i_ on the nucleoprotein filament, and E_het_ if the sites are heterologous. The quadratic factor at the end of the term is an en energy penalty imposed when a dsDNA site tends to slide away from the ssDNA site to which it is bound. It makes sure that bound sites essentially remain in register while still allowing for a certain flexibility. For example, this factor is equal to1 if the sites are perfectly aligned; 0 if their centres are shifted by 0.7*a (approximately one half the nucleoprotein filament size); and < 0 if they are even more displaced. The factor α(l_i_) ensures that there is a penalty for each bond when the duplex site is in an extension state far from optimal 1.5 value. Finally, E_rep_ is a penalty for every binding, which is compensated only if near to optimal conditions are combined: homology, proper alignment of the site, and extension close to 1.5. The value E_rep_ can be regarded as an entropy cost.

Cooperativity cost for binding: every frontier between bound and unbound sites of the dsDNA is penalized, because it is expected to involve a specific distortion of the DNA structure.
