# Supplementary figures and images for: Optimizing the Design of Oligonucleotides for Homology Directed Gene Targeting
Source: PLoS One. 2011 Apr 5;6(4):e14795. doi: 10.1371/journal.pone.0014795 (PMC3071677; doi:10.1371/journal.pone.0014795)

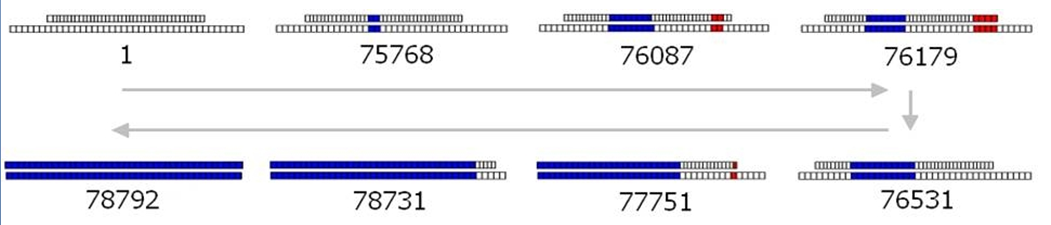

Supplement: Figure S1 — Excerpts of movie 1 showing the local search for homology between a dsDNA and a nucleoprotein filament. The molecules are 45 bases long. Each base of the molecules is represented by a rectangle, as in Fig. 1. The bases are represented in white when they are not in register, in red when they are in register with a non homologous base and in blue when they are in register with a homologous base. (0.13 MB PNG) [file pone.0014795.s002.png]

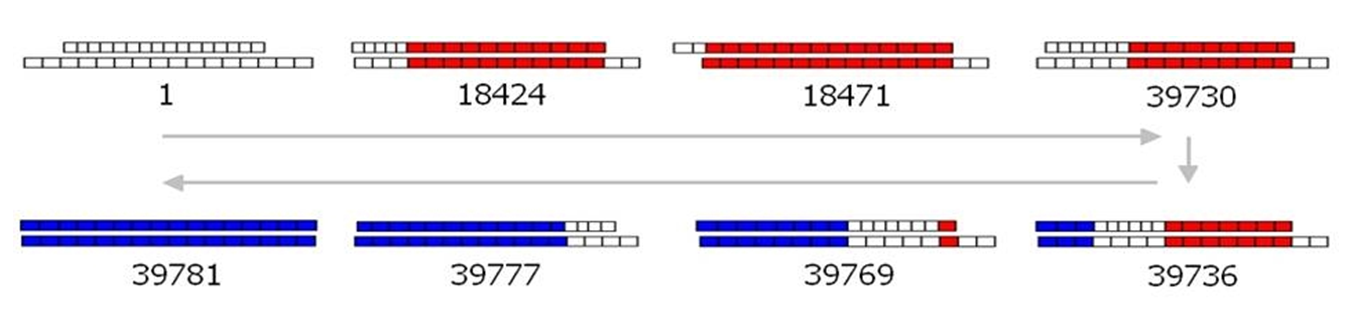

Supplement: Figure S2 — Extracts of movie 2 showing the local search for homology between a dsDNA and a nucleoprotein filament. The molecules are 25 bases long. We used the same colour codes as Fig S1. (0.24 MB PNG) [file pone.0014795.s003.png]
